# The association between persistent cognitive difficulties and depression and functional outcomes in people with major depressive disorder

**DOI:** 10.1017/S0033291722003671

**Published:** 2023-10

**Authors:** F. Matcham, S. K. Simblett, D. Leightley, M. Dalby, S. Siddi, J. M. Haro, F. Lamers, B. W. H. J. Penninx, S. Bruce, R. Nica, S. Zormpas, G. Gilpin, K. M. White, C. Oetzmann, P. Annas, J. C. Brasen, V. A. Narayan, M. Hotopf, T. Wykes, for the RADAR-CNS consortium

**Affiliations:** 1 The Institute of Psychiatry, Psychology and Neuroscience, King's College London, London, UK; 2 School of Psychology, University of Sussex, Falmer, UK; 3 Muna Therapeutics, Copenhagen, Denmark; 4 Parc Sanitari Sant Joan de Déu, Fundació San Joan de Déu, Universitat de Barcelona, CIBERSAM, Barcelona, Spain; 5 Department of Psychiatry, Amsterdam UMC location Vrije Universiteit Amsterdam, Boelelaan 1117, Amsterdam, The Netherlands; 6 Amsterdam Public Health, Mental Health Program, Amsterdam, The Netherlands; 7 The Romanian League for Mental Health, Bucharest, Romania; 8 EPIONI Greek Carers Network, Athens, Greece; 9 H. Lundbeck A/S, Copenhagen, Denmark; 10 Janssen Pharmaceutica NV, New York, USA; 11 South London and Maudsley NHS Foundation Trust, London, UK; 12 www.radar.cns.org

**Keywords:** Cognitive function, epidemiology, longitudinal, major depressive disorder, predictive, remote measurement

## Abstract

**Background:**

Cognitive symptoms are common during and following episodes of depression. Little is known about the persistence of self-reported and performance-based cognition with depression and functional outcomes.

**Methods:**

This is a secondary analysis of a prospective naturalistic observational clinical cohort study of individuals with recurrent major depressive disorder (MDD; *N* = 623). Participants completed app-based self-reported and performance-based cognitive function assessments alongside validated measures of depression, functional disability, and self-esteem every 3 months. Participants were followed-up for a maximum of 2-years. Multilevel hierarchically nested modelling was employed to explore between- and within-participant variation over time to identify whether persistent cognitive difficulties are related to levels of depression and functional impairment during follow-up.

**Results:**

508 individuals (81.5%) provided data (mean age: 46.6, s.d.: 15.6; 76.2% female). Increasing persistence of self-reported cognitive difficulty was associated with higher levels of depression and functional impairment throughout the follow-up. In comparison to low persistence of objective cognitive difficulty (<25% of timepoints), those with high persistence (>75% of timepoints) reported significantly higher levels of depression (*B* = 5.17, s.e. = 2.21, *p* = 0.019) and functional impairment (*B* = 4.82, s.e. = 1.79, *p* = 0.002) over time. Examination of the individual cognitive modules shows that persistently impaired executive function is associated with worse functioning, and poor processing speed is particularly important for worsened depressive symptoms.

**Conclusions:**

We replicated previous findings of greater persistence of cognitive difficulty with increasing severity of depression and further demonstrate that these cognitive difficulties are associated with pervasive functional disability. Difficulties with cognition may be an indicator and target for further treatment input.

## Introduction

Cognitive impairments in major depressive disorder (MDD) include deficits in working memory, attention, executive function, and processing speed, which potentially contribute to low mood, anhedonia, and psychomotor retardation (Rock, Roiser, Riedel, & Blackwell, 2014) and, for some, these difficulties persist into periods of remission (Bora, Harrison, Yücel, & Pantelis, 2013). Self-reported cognitive problems persistent, with studies showing residual cognitive complaints in up to 44% of primary care cases, even when depression has improved (Conradi, Ormel, & De Jonge, 2011). Importantly, persistent cognitive difficulties are associated with a range of negative outcomes, including psychosocial impairment, absenteeism, poor quality-of-life, and a reduced chance of reaching recovery or remission (Atique-Ur-Rehman & Neill, 2019; Baune & Renger, 2014; Ebert et al., 2017; Martinez-Aran et al., 2009).

Despite the prevalence, persistence, and implications of cognitive difficulties in MDD, only 38% of psychiatrists report using cognitive assessments to regularly monitor their patients, or guide treatment decision-making (Belgaied et al., 2014). A challenge for routine assessment is self-report bias (Nieto, Robles, & Vazquez, 2020). Systematic reviews highlight that negative biases in perception, memory, and attention for emotional information, in people with MDD, contribute towards unreliable reporting (Miskowiak & Carvalho, 2014). Baune et al. (2018) also highlight the tendency for people to over-report cognitive function when asked explicitly. Current methods of determining the persistence of objectively-measured cognitive challenges are usually laboratory-based tasks, which may lack ecological validity and require too much resource for frequent repeat testing in large clinical populations (Abramovitch, Short, & Schweiger, 2021).

Identification of cognitive difficulties is critical for the development of treatments for cognitive dysfunction so rapid, objective, valid assessments conducted in naturalistic environments may overcome challenges experienced in the clinic and provide better measures of persistence of difficulties over time. Development of cognitive tests which can be conducted at home, may also alleviate the effects of being observed on cognitive performance and result in more accurate test results (De Carvalho Filho & Yuzawa, 2010). Brief, objective cognitive measures will also allow for the examination of cognitive modules (executive function, working memory, attention, processing speed) that may be relevant for depression and function outcomes (Atique-Ur-Rehman & Neill, 2019).

The Remote Assessment of Disease and Relapse – Major Depressive Disorder (RADAR-MDD; Matcham et al., 2019) study is a multicentre longitudinal observational cohort study in people with a history of recurrent MDD. The project aimed to determine predictors of relapse identifiable via remote measurement technologies including wearable devices and smartphone sensors, however included subjective and objective cognitive assessments collected regularly over an average of 18 months of follow-up. The RADAR-MDD data provides an opportunity to understand the relationship between persistence of cognitive difficulties and changes in depression and functional outcome. The current paper aims to:

1. Describe the persistence of cognitive difficulties in people with MDD.
2. Examine whether there is a relationship between subjective and objective measures of these difficulties.
3. Explore the associations between persistent cognitive difficulties and depression and functioning outcomes.
4. Examine associations between different modules of cognitive difficulties and functional outcomes.

## Methods

### Design

This is a secondary analysis of a prospective observational clinical cohort study: RADAR-MDD (Matcham et al., 2019). RADAR-MDD enroled 623 individuals with recurrent MDD. Participants completed scheduled app-based self-reported and performance-based measurements of cognitive function. These remote assessments were collected alongside validated outcome assessments of depression and physical function every 3 months. Participants were followed-up for a median of 541 days (Matcham et al., 2022b).

### Participants

Participants were aged over 18 years old with a lifetime history of recurrent MDD from the Netherlands, Spain, and UK and had at least two previous episodes and one in the 2 years prior to study entry. To be eligible, participants needed to be able to give informed consent, be fluent in English, Dutch, Catalan, or Spanish, and willing to use an Android smartphone for the duration of follow-up. Exclusion criteria included: a history of bipolar disorder, schizophrenia, MDD with psychotic features or schizoaffective disorder; a diagnosis of dementia; or a major medical disease which might affect the patient's ability to participate in normal daily activities for more than 2 weeks (Matcham et al., 2019).

### Measures

#### Independent variables

*‘Subjective’ cognitive difficulty:* The 5-item Perceived Deficits Questionnaire (PDQ-5) collected self-reported cognitive difficulty, and was administered every 6-weeks via the THINC-it^®^ smartphone app. The PDQ-5 asks respondents to rate how often during the past 7 days they have experienced difficulties with organisation, concentration, and forgetfulness on a scale from 0 (‘Never’) to 4 (‘Often’). The PDQ-5 has been shown to have good reliability (Harrison et al., 2018), and higher scores on the questionnaire indicate higher levels of self-reported cognitive difficulty.

*‘Objective’ cognitive difficulty:* Performance-based measures of cognition were administered every 6-weeks using the THINC-it^®^ smartphone app. The test battery includes four objectively measured cognitive modules. Attention was assessed via the ‘Spotter’ task, which uses the measurement of mean latency for correct responses. Higher scores represent an increased delay in accurately responding, therefore indicating poorer cognitive function. Working memory was assessed using the ‘Symbol Check’ task, providing a total number of correct responses to indicate level of cognitive performance. Higher scores represent increased cognitive function. Processing speed was assessed vie the ‘Code Breaker’ task, which uses the total number of correct responses to represent cognitive performance. Higher scores represent increased cognitive function. Finally, attention switching was measured with the ‘Trails’ task, which provides output describing reaction time in minutes. Higher scores on this module indicate that a longer amount of time was needed to respond, and therefore reduced cognitive performance. For ease of interpretation, PDQ-5 scores, objective cognitive difficulty, Spotter and Trails scores were reversed in the analysis, so that for all modules, higher scores indicate increased cognitive difficulties.

All THINC-it^®^ tasks have validated against paper and pencil versions (McIntyre et al., 2017) and are sensitive to change (Dalby, Annas, & Harrison, 2022; McIntyre et al., 2020). In addition to cognitive domain scores, subscales can be standardised and combined to create a composite score of overall cognitive function (Cha et al., 2017), with higher scores representing increased cognitive difficulties. Previous validation work in MDD has suggested that scores of ⩾1 Standard Deviation (s.d.) below healthy control standardized means from healthy controls can indicate cognitive difficulties (McIntyre et al., 2017).

*Persistence of cognitive difficulty:* Persistence of cognitive difficulty for each measurement of cognitive performance (PDQ-5, composite objective cognitive score, objective module scores) were calculated by creating: (i) whether the individual scored ⩾1 s.d. below standardized means reported in a healthy population (McIntyre et al., 2017) at each timepoint; (ii) the percentage of times the individual scored ⩾1 s.d. below healthy control means; and (iii) quantiles from these percentages resulting in mutually exclusive subcategories for the PDQ-5 and each objective cognitive domain (<25% of all timepoints; 25–50% of all timepoints; 51–75% of all timepoints; and >75% of all timepoints). Participants needed to have completed the relevant assessment at least twice throughout the duration of follow-up for persistence to be included in the analysis.

### Dependent variables

#### Depression symptoms

Scores on each of the 30 items of the Inventory of Depressive Symptomatology-Self Report (IDS-SR) measure (Rush, Carmody, & Reimitz, 2000) were summed to create a total score ranging from 0 to 84, with higher scores indicating higher depression symptom severity and was completed every 3 months. The IDS-SR is well-validated across all languages used in the RADAR-MDD study (Gili et al., 2011; Wardenaar et al., 2010).

#### Functioning

The Work and Social Adjustment Scale (WSAS) (Mundt, Marks, Shear, & Greist, 2002) measures functioning in five domains: work, home management, social leisure, private leisure and personal or family relationships, each scored on a scale of 0–8 with higher scores indicating more disability. Domain scores can be used in isolation or summed to create a total score ranging from 0–40 with higher scores denoting higher disability. The WSAS was completed every 3-months and is well-validated across all languages used in the RADAR-MDD study (Vazquez Morejon et al., 2021; Slagboom et al., 2021).

### Context variables

#### Demographic factors and self esteem

Age, gender, years of education and self-esteem are known to mediate cognitive function and mood (Knight, Rastegar, & Kim, 2016; Santos et al., 2014; Simpson, Hillman, Crawford, & Overton, 2010) so were controlled for in the analyses. Participants' age, gender, and years of education were collected at baseline, and self-esteem using the modified Rosenberg Self-Esteem Scale (RSES) (Greenberger, Chen, Dmitrieva, & Farruggia, 2003). The RSES was collected every two weeks and we used the total score with higher scores representing better self-esteem. Repeated RSES measures were pooled over time in the analysis to adjust for longitudinal change in self-esteem.

### Patient and public involvement

The study was co-developed with service users in our Patient Advisory Board. They were involved in the choice of measures, the timing and issues of engagement and have also been involved in developing the analysis plan and representatives are authors of this paper and have critically reviewed it.

### Data analysis

All data were analysed using STATA (v17.0). First, we tested whether there were systematic differences between those providing and not providing data for analysis using logistic regression. Cross sectional associations between subjective and objective measures of cognitive difficulty were explored using Spearman's correlational analysis. Associations between the cognitive difficulty persistence and time-varying depression or functioning including individual functioning domains were examined using multilevel longitudinal models, pooling data across all 9 timepoints (baseline, 3-months, 6-months, 9-months, 12-months, 15-months, 18-months, 21-months, 24-months). Multilevel models handle hierarchically nested data and can account for between- and within- participant variation over time and missing data (Twisk, de Boer, de Vente, & Heymans, 2013). The main output from the models is the unstandardised maximum likelihood estimates (B coefficients), which provide an estimate of the magnitude and direction of change in depression or functioning according to a reference group (in this case, people with the least cognitive difficulty persistence). Random intercept and time slopes allowed variation in baseline IDS-SR and WSAS scores and rate of change between individuals. Models were adjusted for variables known to influence cognition including age, gender, number of years in education and pooled RSES self-esteem and included time (0 to 24 months) as a continuous variable. Cognitive difficulty persistence was included as a potential categorical predictor to indicate the change in each outcome that was associated with a centile increase of persistence. Linear trends were tested by running separate models with persistence of cognitive difficulty centiles (for both objective and subjective measures) as continuous variables.

To adjust for potential multicollinearity, sensitivity analyses were conducted replicating the above procedure using a modified version of the IDS-SR total score which omits the item on the IDS-SR which measures concentration and decision making.

## Results

### Sample characteristics

A full description of the sample, recruitment and retention rates are available in Matcham et al. (Matcham et al. 2022b). A total of 492 (78.9%) individuals responded to the PDQ-5 at least twice and 448 individuals (71.9%) provided objective cognitive difficulty scores at least twice and were included in the current analysis. In total, the PHQ-5 was completed 4564 times, the Spotter 2872 times, Symbol Check 2871 times, Code Breaker 2838 times and the Trails 2927 times. The median number of THINC-IT^®^ assessments was 10 (IQR: 4–18). The median duration of participation was 539 days (IQR: 407.5–730). There was no apparent association between the total number of THINC-it^®^ assessments and depression severity (*r* = 0.01, *p* = 0.658) or functional ability (*r* = 0.01, *p* = 0.460). Table 1 shows the baseline demographics and clinical characteristics for the entire cohort, and stratified by persistence quantile. In comparison to those with no persistent cognitive difficulties, those with more persistent subjectively reported cognitive difficulties were significantly older, with more severe depression, lower self-esteem, and increased functional disability. They also reported poorer levels of attention, processing speed and working memory. In comparison to those with no persistent cognitive difficulties, those with persistent objectively reported cognitive difficulties were significantly older. Those in the highest persistence group had significantly less years in education, higher depression scores, and more severe impairment in attention, executive function, processing speed and working memory.

**Table 1.** Sample characteristics

		Subjective cognitive difficulty quantiles (PDQ5; *N* = 492)				Objective cognitive difficulty quantiles (THINC-it^®^ Cognitive difficulty composite score; *N* = 448)			
	Total sample (*N* = 623)	1 (ref.) (*N* = 247)	2 (*N* = 61)	3 (*N* = 90)	4 (*N* = 94)	1 (ref.) (*N* = 178)	2 (*N* = 49)	3 (*N* = 36)	4 (*N* = 185)
Age: Mean (s.d.)	46.4 (15.3)	48.2 (15.8)	44.9 (16.6)	43.7 (14.4)*	45.1 (14.9)	35.1 (13.3)	46.7 (13.9)***	52.3 (12.3)***	55.6 (10.8) ***
Female gender, *N* (%)	471 (75.6)	184 (74.5)	46 (75.4)	73 (81.1)	76 (80.9)	141 (79.2)	41 (83.7)	30 (83.3)	134 (72.4)
Education years: Mean (s.d.)	16.4 (6.5)	16.5 (6.6)	15.6 (6.5)	17.1 (7.3)	15.8 (6.3)	17.7 (5.4)	16.1 (6.2)	17.1 (9.8)	15.4 (7.0)**
IDS-SR: Mean (s.d.)	31.3 (14.5)	24.9 (11.4)	36.9 (14.3)*	37.2 (13.8)*	39.0 (14.7)*	28.8 (12.9)	30.4 (13.2)	33.2 (16.3)	33.3 (15.6)**
RSES: Mean (s.d.)	18.2 (3.9)	19.2 (3.4)	16.7 (3.2)*	16.6 (3.6)**	17.1 (4.1)**	17.8 (3.7)	18.7 (2.5)	16.8 (4.8)	18.5 (3.9)
WSAS: Mean (s.d.)									
Total score	19.3 (11.1)	15.7 (10.2)	22.6 (11.7)***	23.7 (9.1)***	23.6 (10.9)***	18.4 (9.4)	18.3 (11.9)	21.1 (11.2)	20.6 (11.7)
Ability to work	3.7 (2.7)	3.1 (2.5)	4.2 (3.0)**	4.7 (2.5)***	4.4 (2.9)***	3.4 (2.3)	3.5 (3.0)	4.1 (2.9)	4.2 (2.9)*
Home management	3.9 (2.6)	3.2 (2.5)	4.3 (2.6)**	4.8 (2.2)***	4.6 (2.4)***	3.8 (2.4)	3.8 (2.6)	3.9 (2.7)	4.0 (2.6)
Social activities	4.3 (2.6)	3.4 (2.4)	5.1 (2.4)***	5.1 (2.8)***	5.4 (2.4)***	4.0 (2.3)	4.2 (2.8)	5.0 (2.6)*	4.5 (2.8)*
Private activities	3.8 (2.6)	3.1 (2.5)	4.7 (2.6)***	4.5 (2.6)***	4.8 (2.4)***	3.6 (2.4)	3.5 (2.6)	4.1 (2.6)	4.1 (2.7)
Relationships	3.6 (2.6)	3.0 (2.4)	4.2 (2.6)**	4.6 (2.2)***	4.5 (2.7)***	3.6 (2.3)	3.3 (2.7)	4.0 (2.6)	3.9 (2.7)
THINC-IT^®^ modules									
PDQ-5: Mean (s.d.)	9.8 (5.1)	6.1 (3.2)	13.9 (3.1)***	13.7 (3.2)***	14.0 (3.2)***	9.1 (4.5)	9.6 (5.4)	9.4 (5.3)	10.2 (5.2)*
Spotter: median latency for correct responses (IQR)	687.0 (569.0–837.0	661.0 (561.0–819.0)	643.0 (522.0–788.0)	721.0 (579.0–845.0)	787.5 (612.0–932.0)***	569.0 (506.0–651.0)	658.0 (575.0–774.0)**	745.0 (661.0–846.0)***	824.0 (697.0–968.0)***
Symbol check: median number correct responses (IQR)	19.0 (13.0–30.0)	20.0 (14.0–31.0)	21.0 (15.0–33.0)	20.0 (12.0–31.0)	16.0 (10.0–24.0)**	31.0 (25.0–36.0)	17.0 (14.0–24.0)***	15.5 (14.5–21.5)***	14.0 (9.0–18.0)***
Codebreaker: median number correct responses (IQR)	47.0 (31.0–61.0)	47.0 (33.0–64.0)	51.0 (37.0–66.0)	47.0 (29.0–60.0)	42.0 (24.0–55.0)**	64.0 (53.0–70.0)	46.0 (35.0–54.0)***	4.0 (36.0–54.0)***	31.0 (19.0–42.0)***
Trails: median minutes taken for completion (IQR)	523.6 (369.5–834.4)	471.7 (342.5–789.3)	506.5 (401.4–758.3)	533.9 (367.0–862.2)	656.1 (468.4–962.8)	371.5 (276.7–455.5)	486.3 (398.7–768.9)	619.8 (412.5–850.1)*	759.5 (533.1–1144.5)***

*p < 0.05 **p < 0.01 *** *p* < 0.001. *p* values ascertained via *t* tests for normally distributed data (age, years in education, IDS, RSES and WSAS data), χ^2^ for gender, and Kruskal Wallis tests for non-normally distributed THINC-it^®^ modules. Persistence quantiles 1 = <25% of timepoints (reference group); 2 = 25–50% of timepoints; 3 = 50–75% of timepoints; 4 = >75% of timepoints).

### Aim 1: The persistence of cognitive difficulties in MDD

**Fig. 1.**
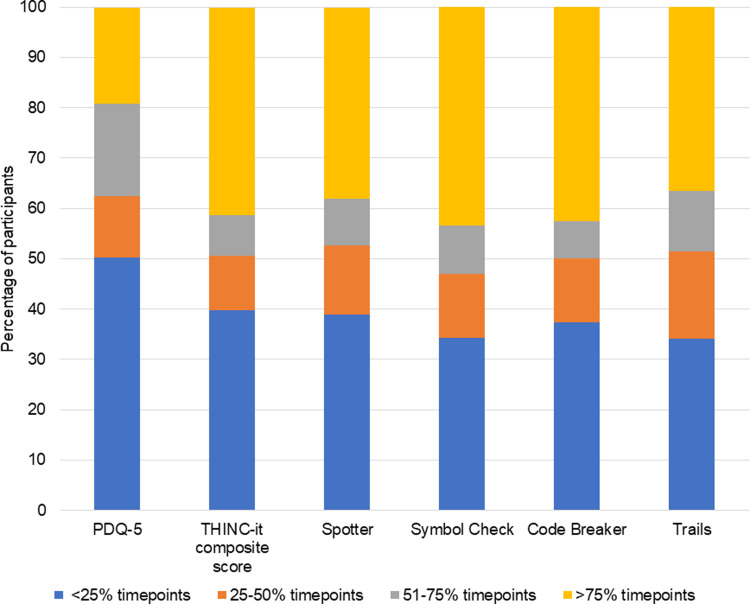
Persistence of cognitive difficulties across assessment modules.

Figure 1 shows the percentage of participants with each level of cognitive difficulty persistence. The lowest persistence appeared in PDQ-5 responses, with nearly 50% of participants self-reporting high levels of cognitive difficulty at less than 25% of timepoints and only 20% at >75% of timepoints. The composite THINC-it^®^ score indicated that an estimated 40% of participants showed signs of cognitive difficulty across modules at >75% of timepoints. Persistence of objective-measured cognitive difficulties were consistent across all modules.

### Aim 2: The relationship between subjectively and objectively measured cognitive difficulties

Table 2 shows the baseline Spearman's correlations between the subjective and objective measures of cognitive difficulties. All objective measures were highly correlated. There were small to moderate associations between subjective and objective measures. Although most comparisons reached the level of statistical significance *p* < 0.05, the strength of the relationship was small.

**Table 2.** Baseline Spearman's correlations between IDS scores, WSAS scores, and subjective and objective measures of cognitive function

	Subjective scores (*N* = 509)			Objective modules (*N* = 448)				
Module	IDS-total	WSAS-total	PDQ-5	THINC-it^®^ composite score	Spotter	Symbol check	Code breaker	Trails
IDS-total	–							
WSAS-total	0.70***	–						
PDQ-5	0.66***	0.55***	–					
THINC-it^®^ composite score	0.21***	0.10*	0.19***	–				
Spotter	0.22***	0.14**	0.23***	0.81***	–			
Symbol check	0.10*	0.17	0.10*	0.84***	0.46***	–		
Code breaker	0.19**	0.08	0.19**	0.88***	0.57***	0.68***	–	
Trails	0.10*	0.03	0.10*	0.54***	0.48***	0.46***	0.57***	–

*p < 0.05, **p < 0.01, ***p < 0.001.

### Aim 3: The association between cognitive difficulties and depression and functioning outcomes

**Fig. 2.**
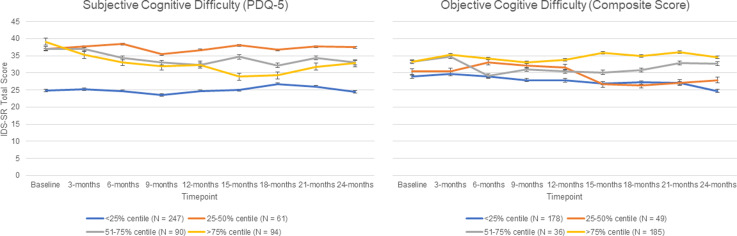
IDS-SR total scores over time, by PDQ-5 subjective cognitive difficulty persistence centile (left panel) and THINC-it^®^ composite score cognitive difficulty persistence centile (right panel). Data shown with standard error bars.

**Fig. 3.**
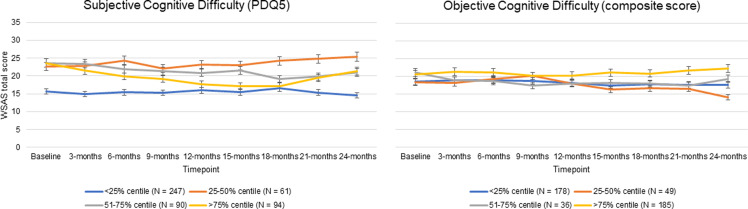
WSAS total scores over time, by PDQ-5 subjective cognitive difficulty persistence centile (left panel) and THINC-it^®^ composite score cognitive difficulty persistence centile (right panel). Data shown with standard error bars.

Results of the adjusted multilevel models (Table 3) and are represented visually in Figs 2 and 3 for depression and function outcomes respectively.

**Table 3.** Associations between elements of cognitive difficulty and depression and functional disability measured throughout follow-up

Cognition domain			IDS-Total			WSAS-total		
Domain	Quantile	*N* (%)	*B* (s.e.)	95% CI	*p* value	*B* (s.e.)	95% CI	*p* value
*Subjective cognitive difficulty*								
PDQ-5 (*N* = 492)								
	1 (<25%; ref)	247 (50.2)	–	**–**	**–**	–	**–**	**–**
	2 (25–50%)	61 (12.3)	**3.69 (1.36)**	**1.02–6.36**	**0.007**	**3.09 (1.06)**	**1.02–5.17**	**0.003**
	3 (51–75%)	90 (18.3)	**3.60 (1.25)**	**1.14–6.10**	**0.001**	**3.55 (0.99)**	**1.62–5.48**	**<0.001**
	4 (>75%)	94 (19.1)	**6.75 (1.61)**	**3.60–9.90**	**<0.001**	**4.32 (1.29)**	**1.80–6.84**	**0.001**
Test for trend		492 (100.0)	**2.14 (0.43)**	**1.30–2.98**	**<0.001**	**1.66 (0.34)**	**0.99–2.33**	**<0.001**
Objective cognitive difficulty								
THINC-it^®^ Cognitive difficulty composite score (*N* = 448)								
		178 (39.7)	–	**–**	**–**	–	**–**	**–**
	1 (<25%; ref)	49 (10.9)	5.12 (2.86)	−0.47 to 10.72	0.073	1.88 (2.28)	−2.58 to 6.34	0.410
	2 (25–50%)	36 (8.0)	3.35 (3.12)	−2.77 to 9.47	0.283	2.80 (2.50)	−2.08 to 7.68	0.262
	3 (51–75%)	185 (41.3)	**7.91 (2.04)**	**2.91–11.91**	**<0.001**	**5.71 (1.65)**	**2.50–8.94**	**0.001**
Test for trend	4 (>75%)	448 (100.0)	**2.47 (0.67)**	**1.16–3.79**	**<0.001**	**1.87 (0.54)**	**0.82–2.93**	**0.001**
Cognition modules								
Attention (Spotter; *N* = 434)		169 (38.9)	–	**–**	**–**	–	**–**	–
	1 (<25%; ref)	60 (13.8)	−1.74 (2.52)	−6.69 to 3.21	0.490	1.61 (2.45)	**−**0.82 to 2.06	0.691
	2 (25–50%)	40 (9.2)	0.02 (2.73)	−5.32 to 5.37	0.993	0.50 (3.23)	−0.87 to 2.22	0.693
	3 (51–75%)	165 (38.0)	**7.04 (1.92)**	**3.27–10.81**	**<0.001**	**8.11 (2.05)**	**3.60–1.57**	**0.022**
Test for trend	4 (>75%)	434 (100.0)	**2.31 (0.64)**	**1.06–3.56**	**<0.001**	**1.14 (0.52)**	**0.13–2.16**	**0.028**
Working memory (Symbol check; *N* = 434)		149 (34.3)	**–**	**–**	**–**	–	–	**–**
	1 (<25%; ref)	55 (12.7)	1.14 (2.58)	−3.92 to 6.20	0.658	1.90 (2.08)	1.90 (2.08)	0.362
	2 (25–50%)	41 (9.5)	0.92 (3.09)	−5.13 to 6.97	0.766	3.49 (2.49)	3.49 (2.49)	0.161
	3 (51–75%)	189 (43.6)	**6.95 (2.13)**	**2.77–11.12**	**0.001**	**4.87 (1.72)**	**4.87 (1.72)**	**0.005**
Test for trend	4 (>75%)	434 (100.0)	**2.32 (0.70)**	**0.95–3.68**	**0.001**	**1.61 (0.56)**	**1.61 (0.56)**	**0.004**
Processing speed (Code breaker; *N* = 434)		162 (37.3)	–	**–**	**–**	–	**–**	**–**
	1 (<25%; ref)	55 (12.7)	0.84 (2.40)	−3.86 to 5.53	0.727	−0.11 (1.96)	−3.95 to 3.73	0.954
	2 (25–50%)	32 (7.4)	0.75 (3.14)	−5.41 to 6.91	0.810	0.51 (2.67)	−4.53 to 5.54	0.844
	3 (51–75%)	185 (42.6)	**8.98 (2.09)**	**4.89–13.07**	**<0.001**	**5.35 (1.71)**	**2.01–8.70**	**0.002**
Test for trend	4 (>75%)	434 (100.0)	**2.93 (0.69)**	**1.58–4.29**	**<0.001**	**1.78 (0.56)**	**0.67–2.88**	**0.002**
Executive function (Trails; *N* = 440)		150 (34.1)	–	**–**	**–**	–	**–**	**–**
	1 (<25%; ref)	76 (17.3)	3.67 (2.06)	−0.36 to 7.71	0.074	1.89 (1.64)	−1.31 to 5.09	0.248
	2 (25–50%)	53 (12.1)	3.92 (2.46)	−0.89 to 8.74	0.110	3.31 (1.96)	−0.53 to 7.15	0.091
	3 (51–75%)	161 (36.6)	**11.68 (20.6)**	**7.63–15.72**	**<0.001**	**7.99 (1.63)**	**4.81–11.18**	**<0.001**
Test for trend	4 (>75%)	440 (100.0)	**3.71 (0.67)**	**2.39 (5.02)**	**<0.001**	**2.61 (0.53)**	**1.58–3.65**	**<0.001**

Models adjusted for age, gender, number of years in education, time and RSES self-esteem at the time of outcome measurement.

Bold text denotes *p* value at level<0.05. *N* = Number of participants.

In comparison to those with the lowest level of persistence of subjective cognitive difficulties (at <25% of timepoints), people with increasing persistence of cognitive difficulties reported higher levels of depression and functional impairment throughout the course of follow-up. For objectively-measured cognitive difficulties, when comparing different persistence centiles with the reference group (<25% of timepoints), only the comparison between the most and least persistent group was significant with those with difficulties over >75% of timepoints having significantly higher levels of depression and functional impairment throughout follow-up.

Analysis of the individual THINC-it^®^ modules highlighted the elements of cognitive performance which consistently impact depression and function outcomes. Highly persistent problems with attention, working memory and processing speed were associated with increased levels of depression and functional impairment throughout follow-up. The largest effect sizes were seen in associations between executive function and depression and functional outcomes. Those with highly persistent (>75% of timepoints) problems with processing speed scored over 11.5 points higher on the IDS-SR throughout follow-up than those with a low level of persistence (<25% of timepoints), and scored 4.5 points higher on the WSAS throughout follow-up than those with a low level of persistence (<25% of timepoints).

Sensitivity analyses excluding the item of the IDS-SR which asks about concentration and decision making did not alter findings (see online Supplementary Table S1).

### Aim 4: The association between cognitive difficulties and different domains of functional outcomes

Higher persistence of subjective cognitive difficulties measured via the PDQ-5 was associated with worse functional impairment across all domains of work and social adjustment (see online Supplementary Table S2). For objectively measured cognitive difficulties, only the highest persistence group showed significant associations across the domains of work and social adjustment.

Increased persistence of attentional difficulty was particularly associated with worse work and private-leisure functioning. Those with the most persistent difficulty with working memory reported worse functioning in work, social-leisure, private-leisure, and relationships over time. Persistent problems with processing speed and executive function were associated with all functional outcomes: work, household, social leisure, private leisure and relationships.

## Discussion

Previous research has demonstrated discrepancies between subjective and objective measures of cognitive difficulty in people with a diagnosis of depression (Petersen, Porter, & Miskowiak, 2019; Srisurapanont, Suttajit, Eurviriyanukul, & Varnado, 2017). We identified strong correlations between the objective THINC-it^®^ modules, but small associations with the subjective PDS-5 measure. Of note, we identified substantially more people with highly persistent objectively-measured cognitive difficulties than self-reported with the PDQ-5, supporting previous suggestions that people may under-report their own cognitive function (Baune et al., 2018). We found a clear relationship between persistent subjective and objective cognitive difficulty and both severity of depressive symptoms and functional impairments in those with a diagnosis of recurrent depressive disorder. For the objective cognitive assessments, we found the largest effect sizes for the most persistently cognitively impaired group. This highlights the potential for at-home smartphone-based cognitive assessments to contribute to identifying those who may be at most risk of poor outcomes.

If we delve into these specific associations further, we see stronger patterns in certain modules. Those with the most persistent difficulties with working memory and executive function appear to rate their functional performance across most functioning domains as lower. Previous research has shown that concentration difficulties account for over 35% of impairment at work (Fried & Nesse, 2014), leaving perhaps also less energy for engagement in leisure activities. While working memory was also associated with reduced engagement in private leisure activities, there was an association with difficulties relating to social leisure activities and relationships too. Differences in brain function during working memory tasks have previously been associated with difficulties in social functioning for people with late onset depression (Pu et al., 2012) and we demonstrate that working memory difficulties affecting social functioning might generalise across a wider range of ages. Persistent difficulties with executive functions and processing speed affected functioning across all domains, suggesting a more pervasive effect on people's lives.

One of the strengths is our novel approach to data collection. We collected multiple cognitive assessments via smartphones, which overcome the time challenges of conventional clinic-based assessments (Matcham et al., 2019). This frequency of assessment allowed us to examine the impact of persistence on outcomes; an often-overlooked concept (Abramovitch et al., 2021). We describe data from an international cohort of individuals with recurrent MDD, so our results have implications across different countries. Of note, we did not find an association between the severity of depression and the number of cognitive assessments completed. This is in line with previous findings that depression severity does not meaningfully impact engagement with remote measurement technologies and emphasises the utility that remote data collection has in even the most severe cases of depression (Matcham et al., 2022a).

The study does have some limitations. Although cognitive assessments may prove a useful predictor of depression and functional status outcomes the THINC-it^®^ tool provided less data for analysis out of all the measurements collected within RADAR-MDD (Matcham et al., 2022b). Due to the data collection infrastructure, it is not possible to determine why data may be missing. We do not know whether technical challenges prevented notifications from being sent, data from being received, or whether people chose not to respond to the assessments. Some patients may be less likely to engage with this method of data collection, and future research would benefit from investigating the barriers to engagement. Ongoing work seeks to identify what may be associated with engagement with the technology and failure to provide data. As yet, our reports have not highlighted any convincing clinical or demographic explanation for loss-to-follow-up (Matcham et al., 2022a), however further work is needed focusing explicitly of cognitive assessments. A further limitation is the lack of causality in our conclusions. We make best use of the data available but cannot determine whether the persistence of cognitive difficulties precedes the trajectories of depression and functionality identified, or if the severity of depression and functional impairment contribute to the persistence of cognitive dysfunction. The most likely relationship is one of bidirectionality (Gonda et al., 2015).

Another consideration is the nature of this study as a secondary analysis of an existing dataset, which was not powered to address this specific question. We used a well-defined threshold of scores ± 1s.d. above/below normal population scores to determine the presence of cognitive difficulties across the cognitive modules, however this often resulted in extremely small group sizes. Our median scores across THINC-it^®^ modules indicate worsened cognitive performance than recently reported in an analysis of healthy controls (Dalby et al., 2022). Although this is expected in a cohort of individuals with long-standing major depression, it means that we often had very small group sizes, increasing our risk of Type 1 error (McCelland, Lynch, Irwin, Spiller, & Fitzsimons, 2015). Our results indicate several large effect sizes which fail to reach statistical significance potentially due to being under-powered.

The limitation of multiple cognitive assessments over long periods of time is the potential for practice effects: the tendency for individuals to perform better over time with repeated opportunities to practice the tasks (Wesnes & Pincock, 2002). Participants were only requested to complete the THINC-it^®^ every 6-weeks, allowing for some standardisation of the duration between assessments across participants. However, our analysis cannot distinguish between the likely differences in performance between those who completed the assessment twice in 2 years, and those who completed it 10 times. Finally, although we have conceptualised the cognitive modules as separate, there is likely to be overlap between the cognition modules (Pan et al., 2019). We attempted to take this into account by creating an overall composite measure of objective cognitive function, but future work may benefit from data reduction techniques to identify the most relevant features.

Our work highlights several recommendations for future investigation. Replicating our findings in case control studies deliberately recruiting individuals with differing levels of cognitive difficulties could ensure comparisons are made across equal groups with sufficient statistical power. We hypothesise that withdrawal from functional activities, particularly social situations, due to difficulties with cognition, may reduce confidence in being able to cope with and get back into, those functional activities. Part of the solution may be increasing coping resources, e.g., through the flexible implementation of cognitive strategies through interventions such as cognitive remediation therapy (Wykes & Reeder, 2006) and aspects of cognitive behavioural therapy focussing on cognitive flexibility (Fazeli, Ehteshamzadeh, & Hashemi, 2015). Also, as the focus on persistence is relatively novel, future research would benefit from attempting to replicate our findings across different measures of cognitive function and using different methods of determining the severity of cognitive difficulties.

## Conclusions

We have demonstrated that when asking people with depression directly about cognitive difficulty there is a relationship between persistent severity of depression and functional disability. We have shown that different elements of cognitive difficulty are differentially associated with worsened depression and function outcomes, with persistent challenges with working memory and executive function most consistently associated with poor outcomes. As we cannot untangle the direction of the relationships further research should explore interventions that target both cognitive and functional disability.

## Supplementary material

For supplementary material accompanying this paper visit https://doi.org/10.1017/S0033291722003671. click here to view supplementary material
