# Supplementary material for: The association between persistent cognitive difficulties and depression and functional outcomes in people with major depressive disorder
Source: Psychol Med. 2022 Dec 13;53(13):6334–44. doi: 10.1017/S0033291722003671 (PMC10520589; doi:10.1017/S0033291722003671)
Supplement: Supplementary file 1 [file S0033291722003671sup001.docx]

Supplementary Table 1. Associations between elements of cognitive difficulty and modified IDS-SR throughout follow-up.

| **Cognition domain** | | |  | | **IDS-Total** | | | |
| --- | --- | --- | --- | --- | --- | --- | --- | --- |
| **Domain** | **Quantile** | **N (%)** | **B(SE)** | | | **95% CI** | **p-value** |  |
| **Subjective cognitive difficulty** | | | |  |  |  |  |  |
| PDQ-5 (N = 492) | 1 (<25%; ref)  2 (25-50%)  3 (51-75%  4 (>75%) | 247 (50.2)  61 (12.3)  90 (18.3)  94 (19.1) | -  **3.44 (1.31)**  **3.42 (1.21)**  **6.25 (1.55)** | | | **-**  **0.89, 6.02**  **1.05, 5.79**  **3.20, 9.29** | **-**  **0.009**  **0.005**  **<0.001** |  |
| *Test for trend* |  | 492 (100.0) | **2.04 (0.43)** | | | **1.18, 2.81** | **<0.001** |  |
| **Objective cognitive difficulty** | | | |  |  |  |  |  |
| THINC-it® Cognitive difficulty composite score (N = 448)  *Test for trend* | 1 (<25%; ref)  2 (25-50%)  3 (51-75%  4 (>75%) | 178 (39.7)  49 (10.9)  36 (8.0)  185 (41.3)  448 (100.0) | -  4.57 (2.77)  **3.39 (3.01**  **7.51 (1.97)**  **2.37 (0.65)** | | | **-**  -0.96, 9.99  -2.51, 9.29  **3.65, 11.37**  **1.11, 3.64** | **-**  0.099  0.260  **<0.001**  **<0.001** |  |
| **Cognition Modules** | | |  | | |  |  |  |
| Attention (Spotter; N = 434)  *Test for trend* | 1 (<25%; ref)  2 (25-50%)  3 (51-75%  4 (>75%) | 169 (38.9)  60 (13.8)  40 (9.2)  165 (38.0)  434 (100.0) | -  -1.37 (2.44)  0.00 (2.63**)**  **6.81 (1.85)**  **2.22 (0.62)** | | | **-**  -6.15, 3.40  -5.15, 5.16  **3.107, 10.44**  **1.02, 3.43** | **-**  0.573  0.999  **<0.001**  **<0.001** |  |
| Working memory (Symbol Check; N = 434)  *Test for trend* | 1 (<25%; ref)  2 (25-50%)  3 (51-75%  4 (>75%) | 149 (34.3)  55 (12.7)  41 (9.5)  189 (43.6)  434 (100.0) | **-**  1.10 (2.50)  0.95 (2.98)  **6.65 (2.06)**  **2.22 (0.67)** | | | **-**  -3.79, 5.99  -4.89, 6.78  **2.62, 10.68**  **0.91, 3.54** | **-**  0.659  0.751  **0.001**  **0.001** |  |
| Processing Speed (Code Breaker; N = 434)  *Test for trend* | 1 (<25%; ref)  2 (25-50%)  3 (51-75%  4 (>75%) | 162 (37.3)  55 (12.7)  32 (7.4)  185 (42.6)  434 (100.0) | -  1.17 (2.31)  0.82 (3.03)  **8.84 (2.10)**  **2.87 (0.67)** | | | **-**  -3.35, 5.70  -5.11, 6.75  **5.90, 12.78**  **1.57, 4.18** | **-**  0.611  0.786  **<0.001**  **<0.001** |  |
| Executive Function  (Trails; N= 440)  *Test for trend* | 1 (<25%; ref)  2 (25-50%)  3 (51-75%  4 (>75%) | 150 (34.1)  76 (17.3)  53 (12.1)  161 (36.6)  440 (100.0) | -  3.55 (1.99)  3.90 (2.37)  **11.22 (1.99)**  **3.56 (*0.65)** | | | **-**  -0.35, 7.44  **-0.74, 8.54**  **7.32, 15.12**  **2.29, 4.83** | **-**  0.074  0.100  **<0.001**  **<0.001** |  |

Models adjusted for age, gender, number of years in education, time and RSES self-esteem at the time of outcome measurement. Bold text denotes p-value at level<0.05. N = Number of participants.

Supplementary Table 2. Longitudinal associations between persistent cognitive difficulty and domains of WSAS functional disability.

| **Domain** | |  |  |  |  | | |  |  |  |  |
| --- | --- | --- | --- | --- | --- | --- | --- | --- | --- | --- | --- |
|  | |  |  | **WSAS – work** | | | | **WSAS -- household** | | | |
|  | | **Quantile** | **N (%)** | **B(SE)** | | **95% CI** | **p-value** | **B(SE)** | | **95% CI** | **p-value** |
| **Subjective Cognitive Difficulty** |  |  |  |  |  |  |  |  |  |  |  |
| PDQ-5 (N = 492)  *Test of trend* | | 1 (<25%; ref)  2 (25-50%)  3 (51-75%  4 (>75%) | 247 (50.2)  61 (12.3)  90 (18.3)  94 (19.1)  492 (100.0) | -  **0.67 (0.28)**  **0.75 (0.26)**  **0.94 (0.33)**  **0.36 (0.09)** | | **-**  **0.11, 1.23**  **0.24, 1.27**  **0.29, 1.60**  **0.18, 0.53** | **-**  **0.019**  **0.004**  **0.005**  **<0.001** | **-**  **0.57 (0.26)**  **1.10 (0.24)**  **0.65 (0.31)**  **0.36 (0.08)** | | **-**  **0.06, 1.09**  **0.61, 1.57**  **0.04, 1.27**  **0.20, 0.53** | **-**  **0.029**  **<0.001**  **0.038**  **<0.001** |
| **Objective Cognitive Difficulty** |  |  |  |  |  |  |  |  |  |  |  |
| THINC-it® Cognitive difficulty composite score (N = 448)  *Test for trend* | | 1 (<25%; ref)  2 (25-50%)  3 (51-75%  4 (>75%) | 178 (39.7)  49 (10.9)  36 (8.0)  185 (41.3)  448 (100.0) | -  0.61 (0.56)  -0.05 (0.61)  **1.12 (0.40)**  **0.34 (0.13)** | | **-**  -0.49, 1.71  -0.25, 1.15  **0.34, 1.90**  **0.09, 0.60** | **-**  0.275  0.933  **0.005**  **0.009** | -  0.62 (0.54)  0.07 (0.59)  **0.96 (0.39)**  **0.29 (0.13)** | | **-**  -0.45, 1.68  -1.09, 1.23  **0.19, 1.72**  **0.04, 0.54** | **-**  0.255  0.906  **0.015**  **0.024** |
| **Cognition Modules** | | | |  | |  |  |  | |  |  |
| Attention (Spotter;  N = 434)  *Test for trend* | | 1 (<25%; ref)  2 (25-50%)  3 (51-75%  4 (>75%) | 169 (38.9)  60 (13.8)  40 (9.2)  165 (38.0)  434 (100.0) | **-**  -0.43 (0.49)  -0.31 (0.54)  **0.92 (0.38)**  **0.30 (0.13)** | | **-**  -1.40, 0.54  -1.36, 0.74  **0.18, 1.66**  **0.05, 0.54** | **-**  0.387  0.560  **0.014**  **0.018** | -  -0.35 (0.49)  0.00 (0.53)  0.64 (0.37)  0.22 (0.12) | | **-**  -1.30, 0.61  -1.03, 1.03  -0.09, 1.37  -0.03, 0.46 | **-**  0.476  1.000  0.084  0.079 |
| Working memory (Symbol Check;  N= 434)  *Test for trend* | | 1 (<25%; ref)  2 (25-50%)  3 (51-75%  4 (>75%) | 149 (34.3)  55 (12.7)  41 (9.5)  189 (43.6)  434 (100.0) | -  0.07 (0.51)  0.36 (0.61)  0.81 (0.42)  **0.28 (0.14)** | | **-**  **-**0.92, 1.07  -0.83, 1.56  -0.01, 1.64  **0.01, 0.55** | **-**  0.888  0.551  0.053  **0.041** | -  -0.06 (0.49)  0.78 (0.59)  0.72 (0.41)  **0.28 (0.14)** | | -  -1.03, 0.90  -0.38, 1.94  -0.09, 1.52  **0.01, 0.55** | **-**  0.895  0.187  0.080  **0.047** |
| Processing Speed (Code Breaker;  N = 434)  *Test of trend* | | 1 (<25%; ref)  2 (25-50%)  3 (51-75%  4 (>75%) | 66 (15.2)  48 (11.1)  40 (9.2)  280 (64.5)  434 (100.0) | -  -0.40 (0.48)  0.23 (0.63)  **0.90 (0.42)**  **0.32 (0.14)** | | **-**  -1.34, 0.53  -1.00, 1.46  **0.09, 1.72**  **0.05, 0.59** | **-**  0.398  0.713  **0.030**  **0.020** | -  0.28 (0.47)  -0.04 (0.61)  **1.05 (0.41)**  **0.33 (0.13)** | | -  -0.64, 1.19  -1.24, 1.16  **0.26, 1.85**  **0.07, 0.60** | -  0.559  0.943  **0.010**  **0.013** |
| Executive Function (Trails; N = 440)  *Test of trend* | | 1 (<25%; ref)  2 (25-50%)  3 (51-75%  4 (>75%) | 150 (34.1)  76 (17.3)  53 (12.1)  161 (36.6)  440 (100.0) | -  0.46 (0.40)  0.74 (0.48)  **1.75 (0.40)**  **0.57 (0.13)** | | **-**  -0.33, 1.26  -0.21, 1.69  **0.96, 2.55**  **0.31, 0.83** | **-**  0.251  0.126  **<0.001**  **<0.001** | -  0.05 (0.40)  0.41 (0.48)  **1.13 (0.40)**  **0.38 (0.13)** | | **-**  -0.73, 0.83  -0.52, 1.35  **0.36, 1.91**  **0.13, 0.64** | **-**  0.892  0.387  **0.004**  **0.003** |

* Models adjusted for age, gender, number of years in education, time and RSES self-esteem at the time of outcome measurement. Bold text denotes p-value at level <0.05.

| **Domain** |  |  |  | | **WSAS – social leisure** | | |  | **WSAS – private leisure** | | |  | **WSAS – relationships** | | |
| --- | --- | --- | --- | --- | --- | --- | --- | --- | --- | --- | --- | --- | --- | --- | --- |
|  | **Quantile** | **N (%)** | **B(SE)** | | | **95%CI** | **p-value** | **B(SE)** | | **95%CI** | **p-value** | **B(SE)** | | **95%CI** | **p-value** |
| **Subjective Cognitive Difficulty** | | | |  |  |  |  |  |  |  |  |  |  |  |  |
| PDQ-5 (N = 492)  *Test of trend* | 1 (<25%; ref)  2 (25-50%)  3 (51-75%  4 (>75%) | 247 (50.2)  61 (12.3)  90 (18.3)  94 (19.1)  492 (100.0) | -  **0.66 (0.27)**  **0.89 (0.25)**  **1.22 (0.31)**  **0.43 (0.08)** | | | **-**  **0.13, 1.18**  **0.41, 1.37**  **0.61, 1.82**  **0.26, 0.59** | **-**  **0.015**  **<0.001**  **<0.001**  **<0.001** | -  **0.90 (0.28)**  **0.76 (0.25)**  **1.17 (0.32)**  **0.41 (0.08)** | | **-**  **0.35, 1.44**  **0.26, 1.26**  **0.54, 1.80**  **0.24, 0.58** | **-**  **0.001**  **0.003**  **<0.001**  **<0.001** | -  **1.00 (0.29)**  0.49 (0.26)  **0.69 (0.33)**  **0.27 (0.09)** | | **-**  **0.44, 1.56**  -0.03, 1.00  **0.05, 1.34**  **0.09, 0.45** | **-**  **<0.001**  0.06  **0.034**  **0.003** |
| **Objective Cognitive Difficulty** | | | |  |  |  |  |  |  |  |  |  |  |  |  |
| THINC-it® Cognitive difficulty composite score (N = 448)  *Test for trend* | 1 (<25%; ref)  2 (25-50%)  3 (51-75%  4 (>75%) | 178 (39.7)  49 (10.9)  36 (8.0)  185 (41.3)  448 (100.0) | **-**  0.41 (0.51)  0.67 (0.56)  1.20 (0.36)  0.39 (0.12) | | | **-**  -0.59, 1.41  -0.43, 1.76  **0.49, 1.91**  **0.16, 0.67** | **-**  0.425  0.233  **0.001**  **0.001** | -  0.61 (0.53)  0.66 (0.58)  **1.16 (0.38)**  **0.28 (0.12)** | | **-**  -0.44, 1.65  -0.47, 1.80  **0.42, 1.90**  **0.13, 0.62** | **-**  0.254  0.253  **0.002**  **0.002** | -  -0.50 (0.53)  1.08 (0.58)  0.86 (0.37)  **0.33 (0.12)** | | **-**  -1.54, 0.53  -0.05, 2.21  **0.13, 1.59**  **0.09, 0.57** | **-**  0.337  0.062  **0.022**  **0.007** |
| **Cognition Modules** | | |  | | |  |  |  | |  |  |  | |  |  |
| Attention  (Spotter; N = 434)  *Test for trend* | 1 (<25%; ref)  2 (25-50%)  3 (51-75%  4 (>75%) | 169 (38.9)  60 (13.8)  40 (9.2)  165 (38.0)  434 (100.0) | -  -0.29 (0.46)  -0.42 (0.50)  0.58 (0.35)  0.18 (0.12) | | | **-**  -1.20, 0.61  -1.40, 0.56  -0.11, 1.27  -0.05, 0.41 | -  0.522  0.400  0.098  0.123 | -  0.30 (0.58)  1.02 (0.61)  **1.03 (0.47)**  **0.24 (0.12)** | | **-**  0.29, 0.48  -0.17, 0.52  **0.81, 0.36**  **0.01, 0.48** | **-**  0.539  0.736  **0.026**  **0.042** | -  -0.03 (0.47)  -0.04 (0.52)  0.64 (0.36)  0.20 (0.12) | | -  -0.96, 0.90  -1.04, 0.97  -0.07, 1.35  -0.03, 0.44 | -  0.951  0.944  0.078  0.088 |
| Working memory (Symbol Check;  N= 434)  *Test for trend* | 1 (<25%; ref)  2 (25-50%)  3 (51-75%  4 (>75%) | 149 (34.3)  55 (12.7)  41 (9.5)  189 (43.6)  434 (100.0) | **-**  0.43 (0.46)  0.62 (0.56)  **1.05 (0.38)**  **0.34 (0.12)** | | | **-**  -0.47, 1.34  -0.47, 1.71  **0.30, 1.80**  **0.10, 0.59** | **-**  0.349  0.268  **0.006**  **0.006** | **-**  0.61 (0.48)  0.82 (0.58)  **1.15 (0.40)**  **0.37 (0.13)** | | **-**  **-0.33, 1.55**  **-0.31, 1.95**  **0.37, 1.93**  **0.12, 0.62** | **-**  0.203  0.157  **0.004**  **0.004** | **-**  0.54 (0.48)  0.71 (0.57)  **1.01 (0.40)**  **0.33 (0.13)** | | **-**  -0.39, 1.47  -0.41, 1.84  **0.24, 1.79**  **0.07, 0.58** | **-**  0.255  0.213  **0.010**  **0.011** |
| Processing Speed (Code Breaker;  N = 434)  *Test of trend* | 1 (<25%; ref)  2 (25-50%)  3 (51-75%  4 (>75%) | 66 (15.2)  48 (11.1)  40 (9.2)  280 (64.5)  434 (100.0) | -  0.22 (0.44)  0.34 (0.57)  **1.23 (0.38)**  **0.40 (0.13)** | | | **-**  -0.64, 1.08  -0.78, 1.47  **0.49, 1.98**  **0.16, 0.65** | **-**  0.618  0.552  **0.001**  **0.001** | -  0.25 (0.45)  -0.41 (0.60)  **1.02 (0.39)**  **0.31 (0.13)** | | **-**  -0.63, 1.14  -1.58, 0.76  **0.25, 1.80**  **0.06, 0.57** | **-**  0.576  0.493  **0.009**  **0.017** | -  -0.59 (0.45)  0.31 (0.59)  **1.02 (0.39)**  **0.37 (0.13)** | | **-**  **-**1.47, 0.28  -0.84, 1.45  **0.26, 1.78**  **0.11, 0.62** | **-**  0.182  0.601  **0.008**  **0.004** |
| Executive Function (Trails; N = 440)  *Test of trend* | 1 (<25%; ref)  2 (25-50%)  3 (51-75%  4 (>75%) | 150 (34.1)  76 (17.3)  53 (12.1)  161 (36.6)  440 (100.0) | **-**  0.38 (0.36)  **0.98 (0.44)**  **1.66 (0.37)**  **0.56 (0.12)** | | | **-**  -0.34, 1.09  **0.12, 1.83**  **0.94, 2.38**  **0.33, 0.79** | **-**  0.304  **0.025**  **<0.001**  **<0.001** | **-**  0.71 (0.38)  0.49 (0.45)  1.77 (0.38)  0.55 (0.12) | | **-**  -0.31, 1.45  -0.40, 1.38  **1.03, 2.52**  **0.30, 0.79** | **-**  0.062  0.284  **<0.001**  **<0.001** | -  0.26 (0.37)  0.63 (0.44)  **1.76 (0.37)**  **0.58 (0.12)** | | **-**  -0.47, 1.00  -0.24, 1.50  **1.03, 2.50**  **0.34, 0.82** | **-**  0.487  0.157  **<0.001**  **<0.001** |

Models adjusted for age, gender, number of years in education, time and RSES self-esteem at the time of outcome measurement. Bold text denotes p-value at level<0.05. N = Number of participants.
